# A Review on Multiscale-Deep-Learning Applications

**DOI:** 10.3390/s22197384

**Published:** 2022-09-28

**Authors:** Elizar Elizar, Mohd Asyraf Zulkifley, Rusdha Muharar, Mohd Hairi Mohd Zaman, Seri Mastura Mustaza

**Affiliations:** 1 Department of Electrical, Electronic and Systems Engineering, Faculty of Engineering and Built Environment, Universiti Kebangsaan Malaysia, Bangi 43600, Selangor, Malaysia; 2 Department of Electrical and Computer Engineering, Faculty of Engineering, Universitas Syiah Kuala, Kopelma Darussalam 23111, Indonesia

**Keywords:** machine learning, artificial intelligence, deep learning, neural network, convolutional neural network, multiscale features

## Abstract

In general, most of the existing convolutional neural network (CNN)-based deep-learning models suffer from spatial-information loss and inadequate feature-representation issues. This is due to their inability to capture multiscale-context information and the exclusion of semantic information throughout the pooling operations. In the early layers of a CNN, the network encodes simple semantic representations, such as edges and corners, while, in the latter part of the CNN, the network encodes more complex semantic features, such as complex geometric shapes. Theoretically, it is better for a CNN to extract features from different levels of semantic representation because tasks such as classification and segmentation work better when both simple and complex feature maps are utilized. Hence, it is also crucial to embed multiscale capability throughout the network so that the various scales of the features can be optimally captured to represent the intended task. Multiscale representation enables the network to fuse low-level and high-level features from a restricted receptive field to enhance the deep-model performance. The main novelty of this review is the comprehensive novel taxonomy of multiscale-deep-learning methods, which includes details of several architectures and their strengths that have been implemented in the existing works. Predominantly, multiscale approaches in deep-learning networks can be classed into two categories: multiscale feature learning and multiscale feature fusion. Multiscale feature learning refers to the method of deriving feature maps by examining kernels over several sizes to collect a larger range of relevant features and predict the input images’ spatial mapping. Multiscale feature fusion uses features with different resolutions to find patterns over short and long distances, without a deep network. Additionally, several examples of the techniques are also discussed according to their applications in satellite imagery, medical imaging, agriculture, and industrial and manufacturing systems.

## 1. Introduction

Automated systems that utilize advanced artificial intelligence technology, and particularly the deep-learning method, have transformed our lives by simplifying various everyday tasks. This technology continues to fascinate us with its limitless opportunities in almost every sector, including e-commerce, healthcare, manufacturing, and entertainment, among many others. It has been extremely successful when applied to imaging input, and it seems to be performing better than humans in a variety of use-case scenarios, and especially in the three most challenging computer-vision applications, which are classification, object detection, and segmentation.

For the image-classification task, the main concern is to obtain the primary labels of all the items that are visible in the image, while, for the object-detection task, the algorithm’s complexity is taken a step further by the attempt to determine the location of the object in the form of a bounding box, in addition to its class. The segmentation task takes the automation challenge to a whole new level by attempting to determine the exact borders of the items in the image. A major category of this task is semantic segmentation, which involves dividing a picture into parts based on its semantic content. It aims to identify the label for each pixel in the image, or, in general, it might refer to segmenting all the pixels in an image into different object categories. It also attempts to split the picture into semantically relevant pieces, and to categorize each portion into one of the predetermined classes, using semantic-segmentation techniques. The same goal can also be achieved by each pixel rather than classifying the entire image. This is what has been defined as a pixel-wise classification, which leads to the same result, but with a slightly different approach. Due to the importance of pixel-wise labeling, there have been various computer-vision projects that rely on semantic segmentation as their foundations. In summary, the objective of semantic segmentation, given an image, is to assign a category label to each pixel [1]. It is a challenging task with many real-world applications, such as autonomous vehicles, sensing technologies, and scene recognition [2]. This semantic-segmentation problem effectively covers both the classification and localization subproblems. However, both these subtasks are inherently conflicting and have a substantial impact on the segmentation model’s design principles. In the case of classification, the models should be invariant to local image details, such as in-plane transformations and deformations, in order to acquire higher-level representations that reflect the global context [3]. This simplification of the spatial information is unfavorable for the localization task, as it requires the model to resolve pixel-level information with good accuracy [4].

It has been proven that the implementation of CNN-based techniques has produced state-of-the-art performances in computer-vision applications for the tasks of classification [5], segmentation [6], object detection [7], and many more. CNN is a model that utilizes feed-forward networks with many hidden layers that are mostly used to extract spatial features for image-processing and object-detection applications [8]. Its hidden layers learn the respective features through a sliding-convolutional-kernel operation, which is usually coupled with an activation function and a pooling layer to produce the best set of feature maps. The last fully connected layer then identifies the object categories. The CNN’s excellent ability to automatically learn the complicated features during the training phase, rather than depending on the handcrafted set of features, is one way in which the segmentation accuracy may well be improved.

CNN feature maps contain spatial variance, which directly implies that they contain spatial dimensions, and as a result, features that are represented by a specific set of feature maps may only become active in a subset of the map’s spatial regions. Apart from the spatial dimensions, this spatial variance also includes spatial scales among the respective entries, at which the localization information of the input images is utilized to estimate the spatial mappings that link them to the input-image regions. Each CNN feature-map activation is only linked to a small number of input units that are all located in the same spatial neighborhood. The spatial-variance characteristic of CNN feature maps arises as a result of the local spatial structure of the convolution filters and the spatially limited receptive fields of their convolution filters. Once these spatial-variance features are extracted, they are then further processed by multiscale processing, and in the end, they improve the ability of the CNN model to obtain a better learning ability.

For the segmentation task, there is also a compromise between the depth of the network and the complexity. However, the reduction in the feature-map size for the classification task will not have too many negative effects, as the goal is to discriminatively identify the class, without concern for the spatial coherency. Hence, if we solely process these deep characteristics, then we will not achieve adequate localization because of the poor resolution.

The most straightforward and efficient way to improve a CNN’s learning capability is to make the network layer deeper. However, this method has several drawbacks, as discussed in [9]:

- There is a tradeoff between the network complexity and processing speed. Typically, a very deep network may produce great accuracy, but it will not be nearly as fast as a lightweight network. This tradeoff applies to both classification and segmentation models;
- If the number of training data is limited, then increasing the network complexity, which directly increases the number of parameters that needs to be fit, will likely result in an overfitting problem;
- The backpropagated gradient will dissipate as the network becomes deeper, leading to the gradient’s diffusion. This circumstance makes it harder to optimize the deep model.

Learning multiscale features, such as multiscale training or changing the receptive field, has resulted in significant performance improvements in the task of image scene classification and segmentation, which appears at different scales due to changes in the image distance and intrinsic object-size properties. This method optimally minimizes the drawback discussed earlier in [9].

The lack of sufficient data to construct a strong training resolution model is one of the challenges that is faced by the existing deep-learning method. Multiscale modeling is highly beneficial because it leverages learning discriminative-feature representation to maximize the information gain and optimize the efficiency by integrating low- and high-resolution data, and by merging multiple sources of data. This nature of multiscale learning has opened up a new paradigm to explain phenomena at a higher scale as a result of the collective action on lower scales. The research presented in [10] suggests a novel method for the early diagnosis of Alzheimer’s disease (AD) by combining multimodal information from MRI and FDG-PET images at multiple scales within the context of a deep neural network. The proposed multiscale approach preserves the structural and metabolic information across multiple scales, extracts features of coarse to fine structures, and increases the diagnostic-classification accuracy.

Multiscale modeling has great flexibility to be combined with another advanced network (such as LSTM, GAN, or another known reference network model) to produce a better performance in classification or segmentation. Parallel configurations of multiscale-deep-learning models and generative networks can be established to provide independent confirmation of the parameter sensitivity. An untapped possibility lies in employing generative models to disentangle the high dimensionality of the parameter variation from the low dimensionality of the dynamics. In one study [11], generative adversarial networks were used to come up with a new method for generalized multiscale feature extraction. The authors used a U-Net architecture with multiscale layers in the generator of the adversarial network to obtain the sequence feature from a one-dimensional vibration signal. The proposed feature-extraction method can accurately predict the RUL, and it outperforms the conventional RUL prediction approaches that are based on deep neural networks.

Moreover, multiscale modeling that consists of multiscale paths is typically implemented at the bottleneck layer due to its smallest low-rank convolution kernel. Thus, this low-rank convolution-kernel approximation is an effective method to reduce the complexity of a deep-learning model, allowing it to classify data more precisely with fewer parameters and less computing effort.

The main contributions of our article are as follows:

1. This is the first comprehensive review on the taxonomy of a multiscale-deep-learning architecture;
2. This review explains, in detail, the two main categories of the multiscale-deep-learning approaches, which are multiscale feature learning and multiscale feature fusion;
3. This is a comprehensive review on the multiscale-deep-learning usage in various main applications, such as satellite imagery, medical imaging, agriculture, and industrial and manufacturing systems.

The originality of our article is based on the comprehensive new insight into the taxonomy of multiscale deep learning. In terms of the application of this multiscale deep learning, it has attracted much interest from many international researchers. We have also provided insight related to the practical perspectives of various multiscale-deep-learning architectures in the form of how they work, as well as their strengths and weaknesses.

The remainder of this article is organized as follows. Section 2 describes the taxonomy of multiscale deep learning that is used in this review paper. Then, in Section 3, various applications of multiscale deep learning in the areas of satellite imagery, medical imaging, agriculture, and industrial and manufacturing systems are described. Finally, Section 4 provides brief concluding remarks on multiscale deep learning.

## 2. Multiscale-Deep-Learning Taxonomy

**Figure 1:**
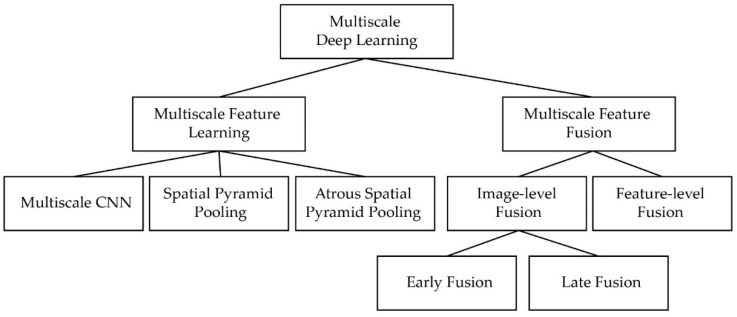
The primary taxonomy of multiscale-deep-learning architecture used in classification and segmentation tasks.

In this section, this review introduces the advanced multiscale-deep-learning methods, primarily for classification and semantic-segmentation tasks. A comprehensive set of taxonomies used in multiscale approaches will be introduced that have been used in the majority of the previous works. Various multiscale-deep-learning architectures will also be discussed, which are broadly classified into two main categories: multiscale feature learning and multiscale feature fusion. This review will help readers to develop a theoretical insight into the design principle of the multiscale-deep-learning network. The main division in the taxonomy of the multiscale-deep-learning architecture is represented in Figure 1.

### 2.1. Multiscale Feature Learning

Recent studies have shown the substantial potential of multiscale learning in various applications, such as scene parsing, self-driving cars, medical diagnosis, and many more [12,13,14]. The underlying idea of multiscale feature learning is to construct several CNN models with various contextual input sizes concurrently, whereby the features from multiple models are combined at the fully connected layer [15]. Multiscale feature learning can be defined as the process of inferring feature maps by analyzing kernels at a variety of scales to capture a wider range of relevant features and estimate the spatial mapping that links to the input images.

**Figure 2:**
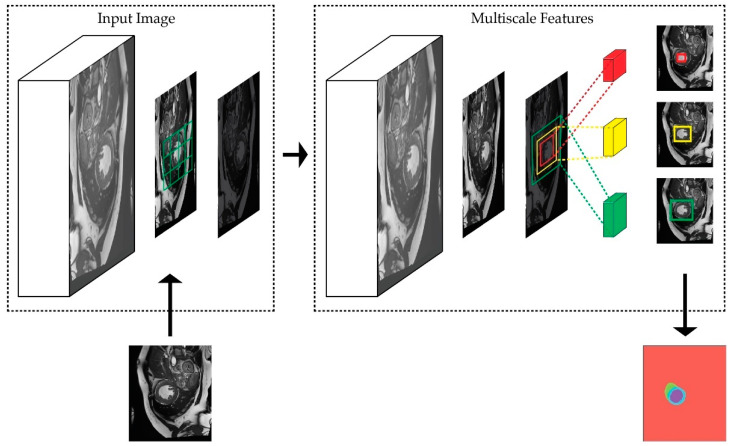
Multiscale receptive fields of deep-feature maps that are used to activate the visual semantics and their contexts. Multiscale representations help in better segmenting the objects by combining low-level and high-level representations.

For a given CNN feature map, the spatial scale of an input image is the size, in pixels, of the rectangle in the input image that affects the value of the respective feature-map registration. Multiscale receptive fields on deep-feature maps aim to capture the semantic and contextual object information, as shown in Figure 2. With respect to the cardiac segmentation example, the red, yellow, and green convolutional filters indicate three different sizes of filters that are used to capture the latent features. The red area tends to be sensitive primarily to the left ventricle, shown in the middle region, while the yellow area covers the endocardium and epicardium regions, and the green area covers the area to the right of the ventricle. The figure also shows that the green area has the largest activation range, which is able to differentiate between the left ventricle, endocardium, and right ventricle with respect to the background information.

#### 2.1.1. Multiscale CNN

**Figure 3:**
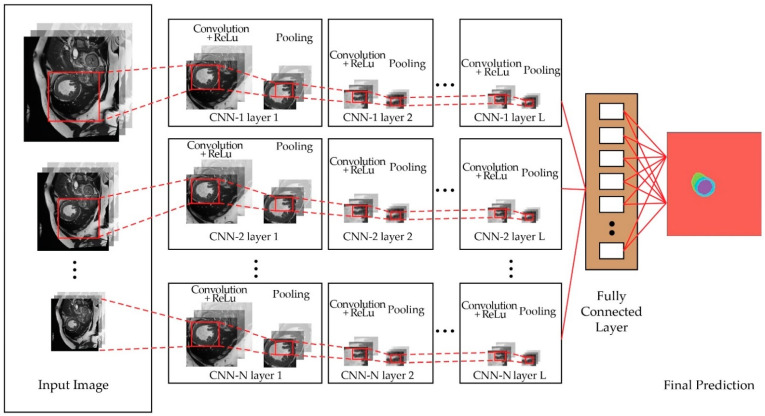
Multiscale CNN, defined as a network with multiple distinct CNN networks with various contextual input sizes that run concurrently, whereby the outputs are combined at the end of the network to obtain rich multiscale semantic features.

The multiscale convolutional method was first introduced in [3] for traffic-sign classification in the German Traffic Sign Recognition Benchmark (GTSRB) competition. Instead of applying strict feed-forward layered designs in a CNN, they used a two-stage convolutional network structured on a parallel path that resembles a dual-network structure. The first-stage output is combined with the second-stage output right before the last fully connected layer. Combining these classifiers that have different input sizes for each parallel path creates different receptive-field widths. Figure 3 illustrates the multiscale CNN structure, which consists of N parallel CNNs with the depth (*L*), and varying contextual sizes of *m* in relation to *N* number of parallel CNNs.

Multiple CNNs may be stacked in parallel to construct a deep CNN, in which the low-level and mid-level features are combined. Let x denote the primary feature maps, $\left\{F_{1},\ldots ,F_{N}\right\}\;$denote all the output channels of the multiscale convolution layer, and $\left\{\left(h_{1},w_{1}\right),\ldots ,\left(h_{m},w_{m}\right)\right\}$ denote the scale factor of the height and width of the feature maps adopted by the m resizing operator. The combination of the *i-*th scale and *j*-th channel yield the *ij-*th multiscale feature maps, as shown in Equation (1):

(1) $$y_{ij}=F_{j}\left(Resize_{h_{i},w_{i}}\left(x\right)\right)$$

When there are *m* resizing operators and *n* different sizes of kernels, the kernels have, at most, *m* × *n* different receptive fields. Multiple resizing operations and different kernel sizes are complementary.

A convolution layer (C^i^) (layer *i* of the network) is influenced by its number (*L*) of convolution maps ($M_{j}^{i}\left(j\in \left\{1,\ldots ,L\right\}\right)$), the kernel size (*K_x_ × K_y_*), and its connection to the previous layer (*L^i^*^−1^). $b_{j}^{i}$ denotes the added bias, and the resultant convolution maps are passed through a nonlinear squashing function, as shown in Equation (2):

(2) $$\emptyset \left(x\right)=1.7159\tan h\left(\frac{2}{3}x\right)$$

The resultant convolution maps are computed as shown in Equation (3) below:

(3) $$M_{j}^{i}=\emptyset \left(b_{j}^{i}+\overset{L}{\underset{n=1}{\sum }}M_{n}^{i-1}*K_{n}^{i}\right)$$

The convolutional layer was followed by a nonlinear activation-function layer, which is the *ReLU* function, as shown in Equation (4):

(4) $$ReLU\left(M_{j}^{i}\right)=\max\left(M_{j}^{i},0\right)$$

The work in [4] constructed four different CNNs that take different input image resolutions with different types of convolution masks, which are then combined in parallel on the same fully connected layer to obtain a vision-based classification of the cells’ application.

The use of pooling in a CNN also enables the network to learn a set of features that are slightly translational- and rotational-invariant, which are desirable properties of natural signals. This kind of multiscale CNN structure was discussed in [4], in which the authors used top-k pooling, where, with a given feature map {*x_i_*}, the top-k pooling defines the result into a single value, as shown in Equation (5):

(5) $$Pool_{k}\left(\left\{x_{i}\right\}\right)=\overset{k}{\underset{r=1}{\sum }}w_{r}a_{r}$$

where $a_{1},\ldots ,a_{k}$ are the highest *k* activations within {*x_i_*}, and $w_{1},\ldots ,w_{k}$ are the nonnegative pooling weights subject to a normalization constraint ($\sum_{r}w_{r}=1$). Exponentially decayed values are used for initialization, and backpropagation is used to optimize the pooling weight.

The resultant feature maps are then fed into the fully connected layer for the classification process over this feature vector.

#### 2.1.2. Spatial Pyramid Pooling (SPP)

**Figure 4:**
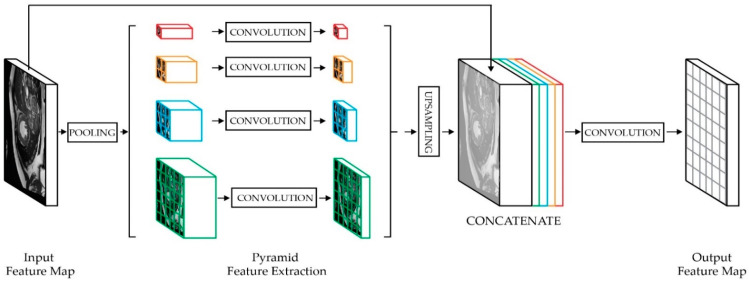
The spatial-pyramid-pooling module extracts information from different scales that varies among different subregions. Using a four-level pyramid, the pooling kernels cover the whole, half, and small portions of the image. A more powerful representation could be fused with information from the different subregions within these receptive fields.

The concept of spatial pyramid pooling (SPP) was initially introduced by He et al. [16]. As shown in Figure 4, SPP involves convolutional layers that are divided into different-sized blocks to extract features with specific dimensions from each size block, which are then fused immediately after the resultant feature map of each parallel path has been resized. Zhao et al. [17] suggested a pooled pyramid structure by using multiple scales of a pooling kernel to combine semantic features of various scales right after a standard decoder network. This inserted module allowed their image-segmentation network to better understand the target context, and to fix the weakness of the fully convolutional network (FCN) [18] in dealing with global information and scenes of various scales. Its pooling-pyramid structure gathers the background information from different areas and keeps the object distortion to a minimum rate. This makes the network better at extracting global information [19].

**Figure 5:**
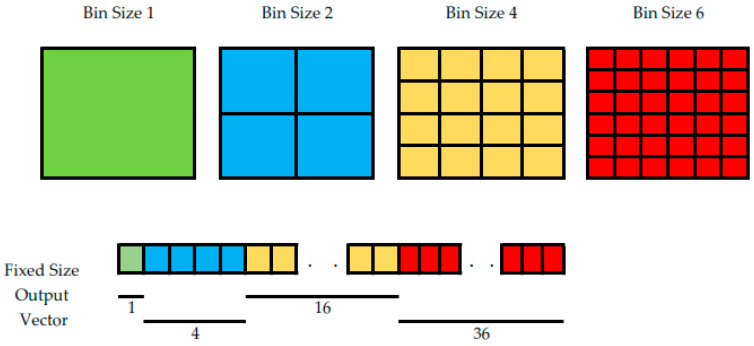
Multilevel spatial bin, with the example of bin-size-6 resultant feature maps segmented into 6 × 6 subsets.

The SPP module is used to replace the conventional pooling approach that is used in CNNs. This pyramid-feature-extraction module makes use of multilevel spatial bins, which split the input feature map into several sections, and then extract features from each of these sections. As shown in Figure 5, any given feature map can be segmented into n × n subsets using bins of size n. In addition, a vector of a fixed size is generated by selecting the largest value from each bin. Each feature map is subsequently pooled numerous times, and its output vectors are concatenated to generate a 1D output vector of feature maps. Assume *n_f_* denotes the number of input feature maps, *n_b_* denotes the number of bins, and *b_i_* denotes the *i*th bins, then the output vector size (*V_s_*) of the SPP is formulated as shown in Equation (6) below [20]:

(6) $$V_{s}=n_{f}*\overset{n_{b}}{\underset{i=1}{\sum }}size{\left(b_{i}\right)}^{2}$$

The SPP technique has been explored and combined with some deep CNN (DCNN) models, whereby it allows the model to generate fixed-length representations, regardless of the image size or image scale of the input features. One of the works that have implemented this technique was designed by the authors of [21], in which they integrated CNN with SPP for the task of vision-based hand-gesture recognition. They also affixed individual SPP operators to each convolutional block to render composite features, which were then fed to a fully connected layer. The other work by Asgari et al. [22] integrated U-Net with an SPP module to create a global feature context, and they applied the model to the task of drusen segmentation for the early detection of age-related macular degeneration (AMD). By using the larger feature context for segmentation, this model outperformed the baseline U-Net model.

#### 2.1.3. Atrous Spatial Pyramid Pooling (ASPP)

Multiscale semantic information plays a crucial role in achieving a high performance for a classification or segmentation network. Hence, atrous spatial pyramid pooling (ASPP) [15,23,24,25] was introduced to capture multiscale contextual information using various dilated convolutions, without any increment to the number of trainable parameters. CNNs with dilated convolution provide alternative capabilities over conventional CNNs, and it is an efficient method to modify the effective receptive field size while maintaining the resolution of the feature maps. Dilated convolutions may be piled on top of one another to produce a series of structures in the same way as ordinary convolution layers can. By piling convolution layers with vastly increased dilation levels, for instance, the receptive field of each convolutional filter can be significantly expanded with just a few additional trainable parameters. Moreover, dilated convolution has been widely explored in various semantic-segmentation tasks [26,27,28]. In order to utilize an effective transfer-learning-initialization method for these segmentation tasks, it is necessary to apply the dilated convolutions sequentially by adding the specified dilated rate to each of the final convolution layers obtained from a pretrained classification network.

For each pixel (*i*) on the output-feature map (*y*) and filter with weight (*w*), atrous convolution is applied to and follows the dilated rate (*r*), as shown in Equation (7) below:

(7) $$y\left[i\right]=\underset{k}{\sum }x\left[i+r.k\right]w\left[k\right]$$

where *y*[*i*] denotes the output of the pixel with index *i*, and *k* denotes the location index in the kernels. The dilated rate determines the stride of the sampling of the input image. The receptive field of the filter can be adjusted by adjusting the rate (*r*).

The ASPP component developed in DeeplabV3 [24] utilizes dilated convolution to capture multiscale information. The ASPP module consists of four parallel atrous convolutions (one 1 × 1 convolution, and three 3 × 3 convolutions with rates of 6, 12, and 18, respectively) and one image global average pooling. The features that are produced by each of the branches are then bilinearly upsampled to the size of the input, concatenated, and put through one more round of 1 × 1 convolution.

**Figure 6:**
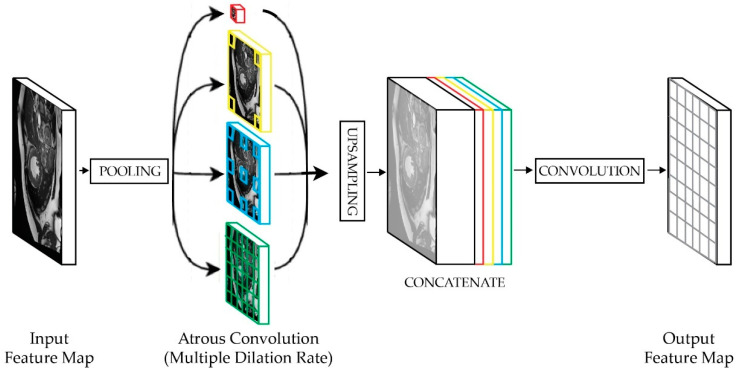
In ASSP, the atrous convolution uses a parameter called the dilation rate that adjusts the field of view to allow a wider receptive field for better semantic-segmentation results. By increasing the dilation rate at each block, the spatial resolution can be preserved, and a deeper network can be built by capturing features at multiple scales.

As shown in Figure 6 below, a set of atrous convolutions is used to address the issue that may be caused by the usage of max pooling, whereby the striding operation deteriorates the network’s resolution. Moreover, ASPP also examines the incoming convolutional feature layer using filters with multiple sampling rates. This approach allows it to capture both the objects and context of the multiscale image, allowing for accurate object separation at multiple scales [29]. The ASPP module ensures that the receptive field is larger by using different dilation rates, arranged in parallel structures. This arrangement is beneficial when processing input with objects and image contexts of various scales [30].

### 2.2. Multiscale Feature Fusion

The goal of a semantic-segmentation model is to predict the semantic class of each individual pixel. Due to this dense prediction requirement, it is typical to maintain high-resolution features so that a better pixel-wise classification can be obtained. However, it is difficult to obtain large receptive fields in high-resolution features by using standard convolution. Multiscale feature fusion is based on the utilization of several features with various resolutions to capture both short- and long-distance patterns, without the need for a very deep network. The multiscale-feature-fusion method is an effective way to obtain high-quality features, which can be divided into image-level fusion and feature-level fusion.

#### 2.2.1. Image-Level Fusion 

**Figure 7:**
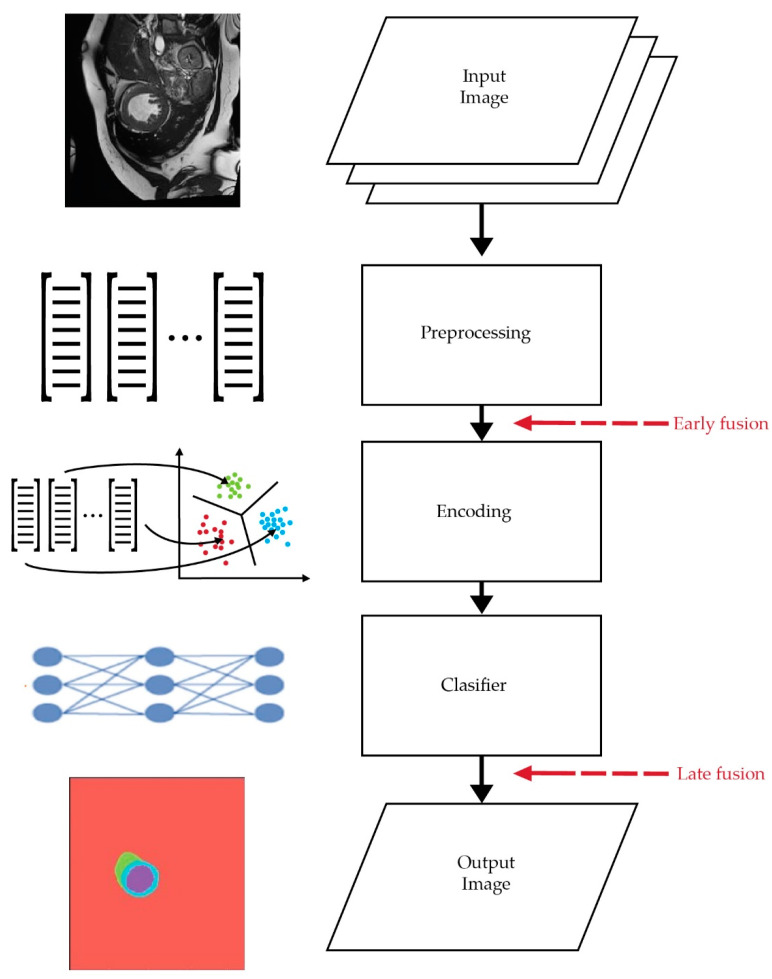
In early fusion, all local attributes (shapes and colors) are retrieved from identical regions and locally concatenated before encoding. In late fusion, image representations are derived independently for each attribute and concatenated afterward.

Image-level fusion is the process of merging important information from multiple images into a single image to produce more useful and comprehensive representations compared with the original inputs. Therefore, by utilizing image-fusion algorithms, the feature-map quality can be enhanced. Based on its feature-fusion state as shown in Figure 7, the image-level-fusion methods can be divided into the following [31,32]:

1. Early fusion: This fusion scheme happens when spatial scales are retrieved from the same regions and concatenated as one input image locally, prior to the encoding. In [33], the authors applied an early-fusion scheme by combining bitemporal remote-sensing images as one input, which was then fed to a modified UNet++ as a backbone to learn the multiscale semantic levels of the visual feature representations for remote-sensing-based change-detection application. Alcantarilla et al. [34] used two bitemporal street-view images that were combined as one input image for their early-fusion method before feeding them into an FCN network to identify the changes in the street-view images;
2. Late fusion: This fusion scheme utilizes separate image representations that are generated for each feature, which are then concatenated together. An example of this late-fusion scheme is the work in [35], which addresses the multiscale problem of the change-detection system by designing a feature-difference network to generate feature-difference maps to provide valuable information at different scales and depths for land-cover application. These learned features are then fed into a feature-fusion network to produce change-detection maps with minimal pixel-wise training samples.

#### 2.2.2. Feature-Level Fusion

The feature-map resolution at the top of the network is relatively poor compared with the bottom layer, and especially around the image boundary, despite its rich semantic information. Meanwhile, the lower feature maps extract low-level semantic features with a higher resolution. Unfortunately, a direct combination of the low-resolution feature maps, as applied in [15,36,37], can only bring about very limited improvements. Due to the repetitive usage of pooling layers in the CNN to extract deeper semantics, the information of small objects can be swept out throughout the downsampling process. The feature-level-fusion method is used to combine high-resolution features with limited semantic information, and low-resolution features with rich semantic information.

**Figure 8:**
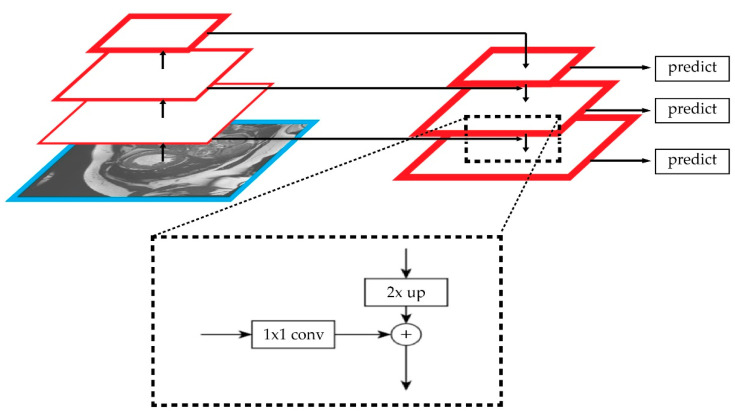
Feature-pyramid-network (FPN) model that combines low- and high-resolution features via a top-down pathway to enrich semantic features at all levels.

One of the commonly used methods for the feature-level scheme is the feature pyramid network (FPN), as proposed in [38]. The FPN aggregates the adjacent feature layer by following Equation (8) below:

(8) $$P_{i}=f_{layer\;i}\left(f_{\;inner\;i}\left(C_{i}\right)+\alpha_{i}^{i+1}*f_{upsample}\left(P_{i+1}^{\prime }\right)\right)$$

where *P_i_* denotes the *i-*th of the feature-map layer; *f_inner i_* denotes the 1 × 1 convolution operation from the bottom-up pathway to reduce the channel dimension; *f_upsample_* denotes the 2× upsampling operation from the top-down pathway for spatial-resolution matching; *f_layer_* denotes a convolution operation for feature processing; α denotes a fusion factor. As shown in Figure 8, the feature maps from the bottom-up pathway and top-down pathway are merged by element-wise addition.

This method improves the feature information by implementing a top-down pathway, and it fuses the features from multiple layers to detect multiscale objects. In the top-down pathways, upsampling operations are used to combine high-level semantic information with low-level spatial information, thereby enhancing the feature characteristics of different levels.

Fan et al. [39] proposed a spatial-attention-based multiscale-fusion module to combine both bottom- and top-layer feature maps. The correlation information between pixels from distinct feature maps is used to solve the semantic gap between scales. The obtained correlations are then used as weight vectors in combining the feature maps.

Wang et al. [40] combines features by adding residual connections and merging receptive fields at multiple scales. The result is a network structure that is composed of many combinations of encoder–decoders that can gather more elaborate properties by combining low-level and high-level semantic information. Furthermore, Wang et al. [41] also employed a multiscale inception block with dilated convolution to construct a high-resolution semantically dense feature extractor during the encoding phase. The multiscale inception block implicitly learns the location information via a large expansivity convolution, while the small expansivity convolution is dedicated to recovering the boundary information. Gao et al. [42] proposed Res2Net, which is an improved version of ResNet [9], by creating residual-like connections with a hierarchical structure within a single residual block. To enhance the size of the receptive field in each layer, a small residual block is added to the initial residual module of the ResNet, which helps the network in extracting multiscale features at the granular level. This model uses the strategy of splitting and fusing the features to allow the convolution operators to process the features more efficiently. Apart from that, using multilevel feature maps could also make the edge information stand out more in the segmentation network. Thus, the negative effect of the low-level features on the high-level semantic features can be lessened by using the pyramidal structure of a neural network during the autonomous search.

## 3. Application of Multiscale Deep Learning

This review will focus on just a few key areas where multiscale deep learning has been used, focusing on where the method has been most widely used in recent studies. This section of the review primarily highlights the application of multiscale deep learning in four different areas, including satellite imagery, medical imaging, agriculture imaging, and multiscale deep learning used in industry and manufacturing.

### 3.1. Satellite Imagery

In recent years, a variety of methods for automating the process of extracting data from satellite images have been developed. These efforts have been applied to a variety of applications with the help of computer-vision algorithms to comprehend the content of satellite images. Some of the applications that have successfully applied intelligent-based remote-sensing systems are agriculture, forestry, urban planning, and climate change research. Historically, satellite imagery is acquired from a bird’s-eye perspective, seen from the top down, and featured in a variety of spectral-band objects. It is represented in multiple channels of a flat 2D plane, which is often at a lower resolution, in which each pixel has its own semantic meaning. Generally, semantic-segmentation models designed for remote-sensing applications aim to extract roads, identify buildings, and classify land cover. The segmentation of satellite images, which is used to find and locate objects and boundaries (straight lines, curves, etc.) of interest in images, is the process of dividing a digital image into several pixel sets. Furthermore, segmentations based on deep-learning techniques have evolved in recent years and have improved significantly with the emergence of fully convolutional neural networks [9].

In some applications of satellite imagery in which the size of the annotated satellite-image datasets is small, for the purpose of semantic segmentation, it is useful to initialize the encoder through the transfer-learning methodology, as in [43,44,45,46], to improve the network performance. Because most satellite images contain objects of varying sizes and shapes, standard DL algorithms with a single-input scale may fail to capture critical scale-dependent characteristics throughout the focal plane. As a result, it is hard to choose the right parameters to form spatial characteristics for various types of objects. Multiscale contextual and compositional elements in the spatial domain should be considered to facilitate the learning process. Zhao et al. [47] proposed a multiscale CNN model that employs dimension reduction, feature extraction, and classification components to construct a pyramid of image models for each component, whereby these models were trained for spatial-analysis purposes. The obtained spatial attributes were then concatenated for the final classification task. Li et al. in [48] combined multiscale CNNs with a bidirectional long short-term memory (Bi-LSTM) network to create spectral-, spatial-, and scale-dependent hyperspectral-image (HIS) attributes. By using this Bi-LSTM, they may take advantage of the correlation among multiscale properties without sacrificing scale-dependent details. Several previous works have focused on observing multiscale CNNs in satellite imagery, and they are summarized in Table 1 below.

### 3.2. Medical Imaging

Deep-learning initiatives are gaining traction in the healthcare domain by bringing about new efficiencies and possibilities that enable physicians, clinicians, and researchers who are passionate about improving the lives of others. Generally, medical imaging is used to diagnose various disorders, including cancer, growth problems, and diabetic retinopathy. Apparently, the use of medical imaging has a huge impact in terms of providing an accurate clinical screening and diagnosis [54,55,56,57,58,59]. One of the subdomains of this application is medical-image segmentation, which uses advanced automated-segmentation algorithms to provide segmentation results that are as similar as possible to the region’s original structure [18,60,61,62]. Deep-learning methods for medical-image applications, either for classification or segmentation purposes, often encounter the following three profound issues:

1. The range of the annotated medical images required for optimally training the model is often limited;
2. The regions of interest (ROIs) are generally small in size, and they have imprecise edges that make them appear in unpredictable x, y, and z positions. Furthermore, sometimes only the entire image label is labeled, even though the targeted ROIs are not available;
3. The ROIs in medical images often contain visual information with similar patterns and that vary in size (scale).

Thus, the multiscale semantic feature plays a crucial role in improving the automation performances of medical-image-classification and segmentation networks. In order to obtain multiscale representations in vision tasks, feature extractors must utilize a wide range of receptive fields to capture the contexts of objects at different scales. Several previous works have focused on observing multiscale CNNs in medical images, and they are summarized in Table 2 below.

### 3.3. Agriculture

Global agricultural production is under increasing amounts of pressure because of several factors, such as population growth, climate change, ecological environment deterioration, the COVID-19 epidemic, and the war in Ukraine. Declining agriculture production could result in severely negative consequences, and especially on food availability, whereby a price hike is to be expected. Therefore, innovative strategies that utilize automated agricultural technology are required to improve the production rates while ensuring sustainable and environmentally friendly farming. The advancements in deep learning, sensor technology, and mechanical automation provide enormous potential to address these challenges.

The adoption of high-performance imaging sensors (RGB, hyperspectral, thermal, and SAR) and unmanned mobile platforms (satellites, drones, and terrestrial robots) is creating huge potential for accurate automated systems. The current use of these imaging data is geared toward transitioning conventional agriculture into data-driven precision agriculture (PA), with the primary objective of reducing the dependency on manual laborious tasks through automation approaches. More importantly, these data contain a large amount of valuable information that will be able to assist farmers in predicting yields, scheduling sowing, tracking the growth states of their crops, monitoring pests and diseases, as well as controlling weeds.

The deep-learning network has been used extensively as part of the automated decision-making tool by extracting hierarchical features from input data for various agricultural tasks. As a result, this wide adoption of DL has opened new possibilities for interpreting massive amounts of data accurately for agriculture analytic systems, such as:

- Crop surveillance systems through remote sensing to map the land cover and crop discrimination [68,69,70];
- Plant-stress-monitoring systems by implementing classification and segmentation networks to better understand the interactions between pathogens, insects, and plants, as well as to determine the causes of plant stress [71,72,73,74,75];
- Disease and pest identification and quantification systems that will assist in monitoring the health condition of plants, including the nutritional status, development phase, and yield prediction [72,76,77,78,79].

Several previous works that have utilized multiscale CNN algorithms in automated agricultural applications are summarized in Table 3 below.

### 3.4. Industrial and Manufacturing Systems

Smart manufacturing makes use of wireless networks, sensors, and intelligent systems to increase the production efficiency, improve the system performance, and reduce wastage while lowering costs [84]. The increasing adoption rate of intelligent sensors and the Internet of things (IoT) has revolutionized the manufacturing sector by allowing computer networks to collect and transform enormous amounts of data from linked machines into information that can be utilized to make automated decisions [85,86]. This has led to an increasing demand for efficient systems to handle high-volume, high-velocity, and high-diversity production data. It happens that the deep-learning approach is able to deliver state-of-the-art analytic capabilities for processing and evaluating massive volumes of production data. It bridges the gap in connecting huge machinery data and intelligent-machine monitoring, which enables it to facilitate the extraction of useful knowledge and make appropriate decisions from vast volumes of data. This approach also promotes high-performance systems through smart manufacturing by reducing the maintenance and operational costs, adapting to customer expectations, boosting productivity, enhancing visibility, and adding more value to the overall operations. Therefore, the deep-learning methodology has contributed to data-driven manufacturing, including the following applications: machine-fault diagnosis, predictive analytics and defect prognosis, and surface-integration inspection.

#### 3.4.1. Machine-Fault Diagnosis

Fault diagnosis is becoming an important step in making mechanical systems safer more reliable. It can prevent large financial losses and injuries to people if the fault can be identified early and accurately. Thus, it is important to ensure that rotating machines, including bearings, gearboxes, and motors, are being observed more accurately so that any problems are found immediately and precisely to prevent potential manufacturing disasters. The conventional approach for fault diagnosis typically includes the manual extraction of the waveform features in order to analyze and process the frequency-domain inputs that are converted from vibration signals [87]. Despite the fact that vibration signals contain a lot of noise, they are the most common type of signal used in fault diagnosis. Before the vibration signal can be used for fault diagnosis, the respective features must be extracted and filtered from the signal to minimize noise [88].

Current findings reveal that the deep-learning-based fault-diagnostic methods are capable of interpreting massive amounts of vibration data to analyze the overall machinery health [89,90,91,92]. However, the classical CNN-based fault-diagnostic methods are not specifically designed for vibration signals, whereby the general convolutional operation typically employs convolutional kernels of the same size, which lacks multiscale feature ability. Different convolutional kernel sizes have specific local reception fields for learning features at different observation scales. This can be used to obtain more useful information from the vibration data, which can be used to improve the fault diagnosis. Jiang et al. [93] proposed a new multiscale architecture by adding a multiscale coarse-grained layer to a standard CNN to extract high-level features using a hierarchical learning framework comprising convolutional and pooling layers. This enables the network to receive complimentary extensive diagnostic information from raw vibration signals. However, the coarse-grained layer is not sufficient enough to fully extract its multiscale information, and that is the reason that Shen et al. [94] combined these layers with the empirical-mode-decomposition (EMD) layer to simultaneously capture the time-domain and frequency-domain features, which further improved the accuracy of the fault diagnosis based on vibration signals.

A deep convolutional network (ConvNet) was proposed for spindle-bearing fault diagnosis by Ding et al. [95], which utilizes wavelet-packet-energy (WPE) images as the input. Instead of relying on vector-characteristic (timeseries) data as an input, a 2D WPE image of the frequency subspaces is reconstructed by using wavelet packet transform to represent the dynamic structure of the WPE distribution for different patterns under varying operating conditions. By doing so, the integral energy and physical relations can be integrated into this particular energy image, whereby the varying features are represented by the differences in the brightness distributions. These distinctive wavelet-packet-energy images (WPIs) are used as the input to train the deep ConvNet architecture. A multiscale-feature-extraction layer, which was used to diagnose the condition of the bearing, was added after the last convolutional layer in order to fully discover the hierarchical representation patterns. This multiscale layer works by implementing a concatenate operator to merge the outputs of the previous pooling layer with the outputs of the most recent convolutional layer.

#### 3.4.2. Predictive Analytics and Defect Prognosis

Bearings are one of the important components that can be found in mechanical systems that may be found rather commonly. Even though they are inexpensive, any failure in the bearings may cause large disruptions to the factory operations, leading to unscheduled downtime and losses in production. Hence, a good bearing-defect-prognosis system is important in reducing the plant downtime and improving the operational safety, as well as for estimating the bearing remaining useful life (RUL), which is necessary for guaranteeing the machinery’s safe operation and reducing maintenance loss. The RUL-estimation methods, using data-driven approaches, attempt to detect the degradation modeling based on the measured data. Statistical tools and machine learning are common data-driven methods to predict the RUL. Furthermore, deep learning is one of the recent machine-learning methodologies with several layers of nonlinear processing elements and convolutional abilities that is able to directly learn the degradation behavior of machinery from unprocessed monitoring data [96,97].

The existing prognostics systems based on the deep-learning approach use sensor data as inputs to the prediction networks in order to combine the full deterioration information of the monitored equipment. However, these methods do not have a clear way to learn how to combine data from different sensors in a useful way. During the designing phase of a network, the engineers assume that each sensor’s input data have an equal impact on the outputs. In real life, however, different levels of degradation can be observed from multiple sensor readings. Some of them might be helpful, but others might not. If there is no way to figure out how different sensors behave and how to highlight the important information on the degradation, then the prediction accuracy of the hidden layers will be affected by irrelevant or redundant information. This will make the RUL predictions of the machinery less accurate and less general. Some multiscale learning strategies have been embedded to automatically extract representations from different temporal scales to combine data from multiple sensors and make RUL predictions more accurate. Using parallel convolutional pathways, Wang et al. [98] created a multiscale technique that allows the prognostics network to automatically collect deterioration signals from multiple temporal scales, and to thus maintain maximum representations.

Zhu et al. [99] suggested a deep CNN-based multiscale bearing-residual-life method. In their network, the hidden layers consist of different-sized convolution kernels that are used to extract both global and local information from the same set of feature maps. The output of the kernels is then linked to the fully connected layer to predict the output value. Jiang et al. [100] proposed a fusion network by combining Bi-LSTM and a multiscale CNN simultaneously in a parallel structure to solve the problem of the long-term dependence on timeseries data, which is a more efficient way of extracting the degradation features to better predict the RUL pattern.

#### 3.4.3. Surface-Integration Inspection

In manufacturing, surface-integration inspections are typically performed with the use of computer-vision and image-processing tools to discover surface defects for the purpose of improving the product quality [101,102]. In this case, the CNN, which was initially developed for image processing, happened to ideally suit the automated defect-identification system of surface-integration inspections.

Several previous works have focused on observing multiscale CNNs in industrial and manufacturing applications, as summarized in Table 4 below.

## 4. Conclusions and Future Works

This work provides a taxonomy for multiscale-deep-learning architectures, and it assesses the current development trends in designing optimal multiscale networks. In this review, multiscale-deep-learning architectures are categorized and discussed according to their usages and applications, which cover the main categories of satellite imagery, medical imaging, agriculture, and industrial and manufacturing systems. After reviewing the strengths and weaknesses of various works regarding the implementation of multiscale deep learning, it is possible to draw the conclusion that multiscale representations have proven to be very significant in improving the classification-, segmentation-, and object-recognition-system performances. Specifically, their performances were enhanced by merging low-level representations from a restricted receptive field with high-level representations to produce a comprehensive and complex set of feature representations.

Given all the previously stated advantages of multiscale-deep-learning methods, this approach still suffers from certain limitations and drawbacks, which are as follows:

- Typically, multiscale networks are constructed by using multiple parallel paths that begin with the coarsest feature map, followed by finer paths, which are progressively added to extract various scale information. The implementation of multiple paths increases the overall network complexity, which directly increases the required computational-resource and memory usage. Due to the volume and resolution of the multiscale data, this method is sometimes impractical for certain applications, and especially for mobile-based systems;
- In order for a multiscale-deep-learning network to be successfully implemented, the emphasis of feature extraction must shift from the global to local scale, allowing the relevance of each connection to be determined at the node level. As a result, the issue creates a challenge in extracting the features as efficiently as possible by combining low-resolution and high-resolution features from different sources. Therefore, the architecture needs to be designed optimally, and as such, the ideal paths for combining the feature maps need to be designed carefully.

Besides this, there are several aspects of multiscale CNNs that can be further explored to improve the general performance of a deep-learning network, such as:

1. Optimal network-flow configurations in the parallel paths. The most popular technique is a direct and homogenous flow scheme for all the parallel paths. A unique network flow, such as a waterfall scheme, can be applied to the parallel paths by cascading down the input between the paths. The second parallel path will receive input from the middle of the first path, and consequently, the third path will receive its input from the middle of the second path. By applying this network flow, the information variation in the input can be expanded while retaining the crucial features of each scale;
2. The optimal implementation of multiple multiscale modules to various selected layers. Usually, a single-feature learning module is added right after the encoder part of a classification network or the bottleneck part of a segmentation network. The main reason for this placement selection is to apply the multiscale module to relatively smaller-size feature maps. However, these reduced feature maps have probably lost some of the crucial information during the downpooling operations, whereby, in a certain network, the multiscale module is applied to small feature maps, which do not carry much information. However, applying the multiscale module at the initial layer will increase the required number of parameters, which directly enlarges the network size and increases the computational workload. Hence, the multiscale module can be selectively applied in the initial and later stages, but with fewer parallel paths for each layer. Hence, the number of parameters can be kept small, but this allows the network to extract multiscale features from various layers;
3. Combining the downsampling approach of SPP and the upsampling approach of ASPP. Usually, in one implementation of the multiscale module, only either SPP or ASPP is applied in the whole network. SPP works by taking multiple-scale input from the downsampling operations, while ASPP work by taking multiple upsamples of the kernel sizes in the atrous convolution. By combining both approaches in one-layer, different types of features can be extracted, as the natures of both modules are different. Therefore, both schemes can be integrated to produce a better multiscale feature extractor; however, the computational workload will also certainly increase.

## Figures and Tables

**Table 1:** The Application of Multiscale Deep Learning in Satellite Imagery.

Literature	Target Task	Network Structure	Method	Strength	Weakness
Gong et al., 2019 [49]	Hyperspectral Image	Spatial Pyramid Pooling	CNN with multiscale convolutional layers, using multiscale filter banks with different metrics to represent the features for HSI classification.	The accuracy is comparable to or even better than other classifications in the both spectral and spectral-spatial classification of the HSI image.	Extracts only the spatial features in the limited-size filtering or convolutional windows.
Hu et al., 2018 [50]	Small Objects	Multiscale-Feature CNN	Identifying small objects by extracting features at different object convolution levels and applying multiscale features.	When compared with Faster RCNN, the accuracy of the small-object detection is significantly higher.	The performance is restricted by the computational costs and image representations.
Cui et al., 2019 [51]	Hyperspectral Image	Atrous Spatial Pyramid Pooling	Integrating both fused features from multiple receptive fields and multiscale spatial features based on the structure of the feature pyramid at various levels.	Better accuracy compared with other classification methods for Indian Pine, Pavia University, and Salina Datasets.	The classification significantly depends on the quality and quantity of the labeled samples, which are costly and time consuming to obtain.
Li et al., 2019 [52]	Aerial Image	Multiscale U-Net	The main structure is U-Net with cascaded dilated convolution at the bottom with varying dilation rates.	The best accuracy for the whole set is compared to four well-known methods using Inria Aerial Image Dataset. The best IoU in Chicago and Vienna Image in the same dataset.	The average IoU performance is still very weak, and especially in the Inria dataset.
Gong et al., 2021 [44]	Hyperspectral Image	Multiscale Fusion + Spatial Pyramid Pooling	The main structure includes a 3D CNN module, a squeeze-and-excitation module, and a 2D CNN pyramid-pooling module.	The method was evaluated on three public hyperspectral classification datasets: Indian Pine, Salinas, and Pavia University. The classification accuracies were 96.09%, 97%, and 96.56%, respectively.	The method still has the misclassification of bricks and gravel. The classification performance is still weak, and especially in the Indian Pine dataset.
Liu et al., 2021 [43]	Hyperspectral Image	Multiscale Fusion	Multiscale feature learning uses three simultaneous pretrained ResNet sub-CNNs, a fusion operation, and a U-shaped deconvolution network. A region proposal network (RPN) with an attention mechanism is used to extract building-instance locations, which are used to eliminate building occlusion.	When compared with a mask R-CNN, the proposed method improved the performance by 2.4% on the self-annotated building dataset of the instance-segmentation task, and by 0.17% on the ISPRS Vaihingen semantic-labeling-contest dataset.	The use of fusion strategies invariably results in increased computational and memory overhead.
Liu et al., 2018 [53]	UC Merced Dataset, SIRI-WHU Dataset, Aerial Image Dataset (AID),	Multiscale CNN + SPP	The proposed method trains the network on multiscale images by developing a dual-branch CNN network: F-net (given that training is performed at a fixed scale), and V-net (given that training is performed with varied input scales per n-iterations).	The MCNN reached a classification accuracy of 96.66 ± 0.90 for the UC Merced Dataset, 93.75 ± 1.13 for the SIRI-WHU Dataset, and 91.80 ± 0.22 for the AID Dataset.	This method reduces the possibility of feature discrimination by focusing solely on the feature map from the last CNN layer and ignoring the feature data from additional layers.
Gao et al., 2022 [46]	Hyperspectral Image	Multiscale Fusion	This method employs cross-spectral spatial-feature extraction (SSCEM). This module sent previous CNN layer information into the spatial and spectral extraction branches independently, and so changes in the other domain after each convolution could be fully exploited.	The proposed network excels in many deep-learning-based networks on three HSI datasets. It also cuts down on the number of training parameters for the network, which helps, to a certain extent, to prevent overfitting problems.	The performance is restricted by the complexity of the network structure, which implies a greater computational cost.

**Table 2:** The Application of Multiscale CNNs in Medical Imaging.

Literature	Target Task	Network Structure	Method	Strength	Weakness
Wolterink et al., 2017 [63]	Vessel Segmentation	CNN + Stacked Dilation Convolution	CNN with ten-layer network. The first eight layers are the feature-extraction levels, whereas Layers 9 and 10 are fully connected classification layers. Each feature-extraction layer uses 32 kernels. The level of the dilation rate increases between Layers 2 and 7.	The myocardium and blood pool had Dice indices of 0.80 ± 0.06 and 0.93 ± 0.02, respectively, average distances to boundaries of 0.96 ± 0.31 and 0.89 ± 0.24 mm, respectively, and Hausdorff distances of 6.13 ± 3.76 and 7.07 ± 3.01 mm, respectively.	Due to hardware limitations, the work still used a large receptive field and led to a less precise prediction.
Du et al., 2020 [64]	Vessel Segmentation	Dilated Residual Network + Modified SPP	The network’s inception module initializes a multilevel feature representation of cardiovascular pictures. The dilated-residual-network (DRN) component extracts features, classifies the pixels, and anticipates the segmentation zones. A hybrid pyramid-pooling network (HPPN) then aggregates the local and worldwide DRN information.	Best result in quantitative segmentation compared with four well-known methods in all five substructures (left ventricle (LV), right ventricle (RV), left atrium (LA), right atrium (RA), and LV myocardium (LV_Myo)).	The HD value of this method is higher than that of U-Net, which shows that it still has some issues with segmenting small targets.
Kim et al., 2018 [45]	Lung Cancer	Multiscale Fusion CNN	Multiscale-convolution inputs with varying levels of inherent contextual abstract information in multiple scales with progressive integration and multistream feature integration in an end-to-end approach.	On two parts of the LUNA16 Dataset (V1 and V2), the method did much better than other approaches by a wide margin. The average CPMs were 0.908 for V1, and 0.942 for V2.	The anchor scheme used by the nodule detectors introduces an excessive number of hyperparameters that must be fine-tuned for each unique problem.
Muralidharan et al., 2022 [65]	Chest X-ray	Multiscale Fusion	The input image is divided into seven modes, which are then fed into a multiscale deep CNN with 14 layers (blocks) and an additional four extra layers. Each block has an input layer, convolution layer, batch-normalization layer, dropout layer, and max-pooling layer, whereby the block is stacked three successive times.	The proposed model successfully differentiates COVID-19 from viral pneumonia and normal classes with accuracy, precision, recall, and F1-score values of 0.96, 0.97, 0.99, and 0.98, respectively.	The obtained results are still based on random combinations of the extracted modes, and so they need to run the model with every possible combination of the hyperparameters to obtain the desired result.
Amer et al., 2021 [66]	Echocardiography	Multiscale Fusion + Cascaded Dilated Convolution	The network uses residual blocks and cascaded-dilated-convolution modules to pull both coarse and fine multiscale features from the input image.	Dice-similarity-performance measure of 95.1% compared with expert’s annotation and surpasses Deeplabv3 and U-Net performances by 8.4% and 1.2%, respectively.	The work only measures the image-segmentation performance, without including the LV-ejection-fraction (ED and ES) clinical cardiac indicators.
Yang et al., 2021 [67]	Cardiac MRI	Dilated Convolution	The dilated block of the segmentation network captures and aggregates multiscale information to create segmentation probability maps. The discriminator part differentiates the segmentation probability map and the ground truth at the pixel level to provide confidence probability maps.	The Dice coefficients on the ACDC 2017 for both ED and ES are 0.94 and 0.89, respectively. The Hausdorff distances for both the ED and ES are 10.6 and 12.6 mm, respectively.	The model still produces weak Dice coefficients in both the ED and ES of the left-ventricle-myocardium part.
Wang et al., 2021 [41]	Cardiac MRI	Multiscale Fusion/Dilated Convolution	The encoder part uses dilated convolution. The decoding part reconstructs the full-size skip-connection structure for contextual-semantic-information fusion.	The Dice coefficients on the ACDC 2017, MICCAI 2009, and MICCAI 2018 datasets reached 96.2%, 98.0%, and 96.8%, respectively. Overall, Jaccard indices of 0.897, 0.964, and 0.937 were observed, with Hausdorff distances of 7.0, 5.2, and 7.5 mm, respectively.	The work only measures the image-segmentation performance, without including the LV-ejection-fraction (ED and ES) clinical cardiac indicators.
Amer et al., 2022 [30]	Echocardiography Lung Computed Tomography (CT) Images	U-Net + Multi-scale Spatial Attention + Dilated Convolution	The model uses a U-Net architecture with channel attention and multiscale spatial attention to learn multiscale feature representations with diverse modalities, as well as shape and size variability.	The proposed model outperformed the basic U-Net, ResDUnet, Attention U-Net, and U-Net3+ models by 4.1%, 2.5%, 1.8%, and 0.4%, respectively, on lung CT images. It also outperformed the basic U-Net, ResDUnet, Attention U-Net, and U-Net3++ models by 2.8%, 1.6%, 1.1%, and 0.6%, respectively, on the left-ventricle images.	The approach still struggles to capture edge details accurately, and it loses segmentation detail at complicated edges.

**Table 3:** The Application of Multiscale Deep Learning in Agriculture Sensing.

Literature	Target Task	Network Structure	Method	Strength	Weakness
Hu et al., 2018 [74]	Plant Leaf	Multiscale Fusion CNN	With a list of bilinear interpolation procedures, the input image is split up into several low-resolution images. These images are then fed into the network so that it can learn to understand different features at different depths.	Produced a better accuracy rate in most of the MalayaKew Leaf Dataset and LeafSnap Plant Leaf Dataset.	The training process required a more complex sample set that needed to provide both whole and segmented images.
Li et al., 2018 [80]	Chinese Herbal Medicines	Multiscale Fusion CNN	Near and far multiscale input images are fused together into a six-channel image using a CNN of three convolutional and three pooling layers.	The requirements of Chinese-herbal-medicine classification were met by the model, with a classification accuracy of more than 90%.	There are still many problems with the method, such as less training data, a less accurate classification, and less ability to avoid interference.
Turkoglu et al., 2021 [70]	ZueriCrop Dataset	Early Fusion + CNN	The model consists of layered CNN networks. In a hierarchical tree, different network levels are indicative of increasingly finer label resolutions. At the refining stage, the three-dimensional probability regions from three different stages are passed to the CNN.	The achieved precision, recall, F1 score, and accuracy are 0.601, 0.498, 0.524, and 0.88, respectively, which outperforms the advanced benchmarked methods.	It is unclear how to adapt the model layout to standard CNNs without affecting the feature-extraction backbone for recurrent networks.
Li et al., 2021 [81]	Crop Image (UAVSAR and RapidEye)	Multiscale Fusion CNN	A sequence of object scales is gradually fed into the CNN, which transforms the acquired features from smaller scales into larger scales by adopting gradually larger convolutional windows.	This technique provides a novel method for solving the issue of image classification for a variety of terrain types.	The model still generates blurred boundaries between crop fields due to the requirement for an input patch.
Wang et al., 2021 [82]	Tomato Gray Mold Dataset	Feature Fusion + MobileNetv2 + Channel Attention Module	MobileNetv2 was used as the base network, whereby multiscale feature fusions provide the fused feature maps. The efficient channel-attention module then enhances these feature maps, and the relevant feature paths are weighted. The resultant features were used to predict mold on tomatoes.	Precision and F1 score reached 0.934 and 0.956, respectively, and it outperformed the Tiny-YOLOv3, MobileNetv2-YOLOv3, MobileNetv2-SSD, and Faster R-CNN performances.	Missed detection persists, and especially at extreme shooting angles, and it imposes inaccurate early diagnosis at different parts under different shooting conditions.
Zhou et al., 2022 [83]	Fish Dataset	ASPP + GAN	A generative adversarial network (GAN) is introduced before applying CNN to augment the existing dataset. Then, the ASPP module fuses the input and output of a dilated convolutional layer with a short sample rate to acquire rich multiscale contextual information.	On the validation dataset, the obtained F1 score, GA, and mIoU reached 0.961, 0.981, and 0.973, respectively.	The model still loses a lot of segmentation detail at the complicated edges.

**Table 4:** The Application of Multiscale Deep Learning in Industrial and Manufacturing Systems.

Literature	Target Task	Network Structure	Method	Strength	Weakness
Ding X., He Q., 2017 [95]	Fault Bearing Dataset	Wavelet-Packet-Energy (WPE) Image + Deep Convolutional Network	The deep convolutional network has three convolutional layers, two max-pooling layers, and one multiscale layer. The multiscale layer combines the final convolutional layer’s output with the subsequent pooling layer’s output to diagnose any issue on the bearing. Six spindle-bearing datasets with ten-class health states under four loads are used to verify the proposed method performance.	The deep convolutional network achieved stable and high identification accuracies of 98.8%, 98.8%, 99.4%, 99.4%, 99.8, and 99.6 for datasets A, B, C, D, E, and F, respectively.	Increased complexity, which implies a greater computational cost and is impractical in practice.
Jiang et al., 2020 [100]	C-MAPSS Dataset	Bi-LSTM and Multiscale CNN Fusion Network	The last 3 layers of the fusion network used Bi-LSTM with 64 cells, a multiscale CNN with 32 convolution kernels, and 2 × 2 maximum pooling kernels. The combined output of the two networks determines the predicted RUL.	The proposed fusion model has better RMSE indicators compared with the CNN, LSTM, and Bi-LSTM, tested on four subsets of the dataset.	The method is prone to overfitting, and it is difficult to use the dropout algorithm to prevent it because recurrent connections to LSTM units are probabilistically removed from the activation and weight updates during network training.
Wang et al., 2021 [103]	Pronostia Bearing Dataset	Multiscale-CNN with Dilated Convolution Block	A complex signal is decomposed using an integrated dilated convolution block. Multiple stacked integrated dilated convolution blocks are fused to create a multiscale feature extractor to mitigate the information loss.	The mean absolute error (MAE) and root mean squared error (RMSE) of the proposed method are the lowest among the comparison methods.	The method does not include uncertainty prediction in the deep-learning model, making it impractical in practice.
Zhu et al., 2019 [99]	Pronostia Bearing Dataset	Multiscale-CNN	The time-frequency representation (TFR) can represent a complex and nonstationary signal of the bearing degradation. The TFRs and their assigned RULs were sent to a multiscale model structure to pull out more features that could be used to predict the RUL. The multiscale layer maintains the global and local properties to boost the network capacity.	The mean absolute error (MAE) and root mean squared error (RMSE) of the proposed method are the lowest among the other data-driven methods.	The performance is restricted by the complexity of the network structure, which implies a greater computational cost.
Li et al., 2020 [104]	C-MAPSS Dataset	Multiscale Deep Convolutional Attention Network	The MS-DCNN has three different sizes of convolution operations and multiscale blocks that are put together in parallel. The three multiscale-block output-feature maps are passed to a standard CNN after the multiscale convolution. At the end of the MS-DCNN network, one neuron is connected to provide the final result of the predicted RUL value.	Compared with the other advanced methods, such as the semi-supervised setup, MODBNE, DBN, and LSTM, the RMSE indicators of the proposed method reduced the error by 8.92%, 14.87%, 3.55%, 1.94%, respectively, tested on four datasets.	To learn the prediction models, the method needs a substantial amount of data, which may not be feasible in real-life situations.
Wang et al., 2021 [98]	Pronostia Bearing Dataset	Multiscale Convolutional Attention Network	First, self-attention modules are constructed to combine multisensor data. Then, an automatic multiscale learning technique is implemented. Finally, high-level representations are loaded into dynamic dense layers for regression analysis and RUL estimation.	The proposed strategy fuses multisensor data and improved RUL-prediction accuracy. Its prediction performance was better than previous prognostics methods.	The approach incorrectly presumes that the monitoring data collected by different sensors contribute equally to the RUL estimation, which leads to an inaccurate RUL prediction.

## Abbreviations

The following abbreviations are used in this manuscript:

CNN	Convolutional Neural Network
ReLU	Rectified Linear Unit
FCN	Fully Convolutional Network
GTSRB	German Traffic Sign Recognition Benchmark
SPP	Spatial Pyramid Pooling
PSPNet	Pyramid Scene Parsing Network
ASPP	Atrous Spatial Pyramid Pooling
FPN	Feature Pyramid Network
ResNet	Residual Neural Network
LSTM	Long Short-Term Memory
Bi-LSTM	Bidirectional Long Short-Term Memory
HSI	Hyperspectral Imaging
2D	Two-Dimensional
3D	Three-Dimensional
SSCEM	Spectral-Spatial-Feature Cross-Extraction Module
RPN	Region Proposal Network
RCN	Region-Based Convolutional Neural Networks
AID	Aerial Image Dataset
ROI	Region of Interest
ACDC	Automated Cardiac Diagnosis Challenge
MICCAI	Medical Image Computing and Computer-Assisted Intervention
ED	End-Diastolic
ES	End-Systolic
DRN	Dilated Residual Network
HPPN	Hybrid Pyramid-Pooling Network
CT	Computed Tomography
RGB	Red Green Blue
SAR	Synthetic-Aperture Radar
PA	Precision Agriculture
GAN	Generative Adversarial Network
mIoU	Mean Intersection over Union
GA	Global Accuracy
UAVSAR	Uninhabited Aerial Vehicle Synthetic Aperture Radar
YOLOv3	You Only Look Once version 3
SSD	Single Shot Detector
IoT	Internet of Things
EMD	Empirical Mode Decomposition
RUL	Remaining Useful Life
WPE	Wavelet Packet Energy
ConvNet	Convolutional Network
MAE	Mean Absolute Error
RMSE	Root Mean Squared Error
TFR	Time-Frequency Representation
MSCNN	Multiscale Convolutional Neural Network
DBN	Deep Belief Network
MODBNE	Multi-Objective Deep Belief Network Ensemble
C-MAPSS	Commercial Modular Aero-Propulsion System Simulation
MS-DCNN	Multiscale Deep Convolutional Neural Network
